# Initiating DNA replication: a matter of prime importance

**DOI:** 10.1042/BST20180627

**Published:** 2019-01-15

**Authors:** Stephen D. Bell

**Affiliations:** 1 Department of Molecular and Cellular Biochemistry, Indiana University, Simon Hall MSB, 212 S Hawthorne Dr., Bloomington, IN 47405, U.S.A.; 2 Department of Biology, Indiana University, Simon Hall MSB, 212 S Hawthorne Dr., Bloomington, IN 47405, U.S.A.

**Keywords:** archaea, DNA replication, primase

## Abstract

It has been known for decades that the principal replicative DNA polymerases that effect genome replication are incapable of starting DNA synthesis *de novo*. Rather, they require a 3′-OH group from which to extend a DNA chain. Cellular DNA replication systems exploit a dedicated, limited processivity RNA polymerase, termed primase, that synthesizes a short oligoribonucleotide primer which is then extended by a DNA polymerase. Thus, primases can initiate synthesis, proceed with primer elongation for a short distance then transfer the primer to a DNA polymerase. Despite these well-established properties, the mechanistic basis of these dynamic behaviours has only recently been established. In the following, the author will describe recent insights from studies of the related eukaryotic and archaeal DNA primases. Significantly, the general conclusions from these studies likely extend to a broad class of extrachromosomal element-associated primases as well as the human primase-related DNA repair enzyme, PrimPol.

## Introduction

As with the rest of the DNA replication machinery, the archaeal DNA primases have an orthologous relationship to their counterparts in eukaryotes and are structurally distinct from the primases encoded in bacterial genomes [1]. The bacterial primase, DnaG, is based on a ‘Toprim’ fold — a module of roughly 100 amino acids that is found in topoisomerases, bacterial primases and several nucleases [2]. Interestingly, most archaeal genomes encode a protein that possesses a Toprim fold and which has been termed DnaG in many genome annotations. However, this protein has been found to be stably associated with the exosome of *Sulfolobus solfataricus* and recent studies have revealed it to be an RNA-binding protein that possesses the ability to effect polynucleotidylation of stable RNA molecules, thereby facilitating their degradation by the exosome [3]. In contrast, the true DNA primase of archaeal species is based on a heterodimeric assembly of two subunits homologous to the PriS and PriL subunits of the eukaryotic primase assembly [4]. The eukaryotic core primase PriSL associates further with DNA polymerase alpha and its accessory factor B-subunit [5]. These latter two proteins are absent from archaea. PriS has an α/β-structure and contains the catalytic site for primer elongation. PriL, often referred to as the regulatory subunit, is largely α-helical and contains a signature iron-sulfur cluster of the 4Fe–4S type [6–9]. The N-terminal half of PriL mediates interactions with PriS, while the C-terminal portion of the protein possesses two subdomains, the most C-terminal of which co-ordinates the FeS cluster. Interestingly, recent studies have indicated that the two subdomains are closely related to one-another, forming a pseudo-tandem repeat [10]. In many archaea, the functional primase appears to be the PriSL heterodimer [11]. However, in the highly studied species of the genus *Sulfolobus*, PriSL associates with a third subunit, PriX, to form a functional heterotrimer [12]. PriX was identified by the laboratory of Li Huang following affinity purification of the endogenous primase complex. Subsequent reconstitution experiments revealed that the addition of PriX massively stimulated the activity of PriSL from its very low basal level. The X-ray crystal structure of PriX in isolation was solved, revealing it to be related structurally to the C-terminal subdomains of PriL, despite an absence of primary sequence conservation [12,13].

Classically, primases can initiate synthesis of RNA *de novo*. Remarkably, several studies of archaeal primases have revealed that they can also initiate DNA synthesis, when supplied with dNTP precursors. This property was confirmed for PriSLX, although, intriguingly, the presence of dNTPs led to a general inhibition of the nucleic acid synthetic ability of primase with an overall reduction in primer length. This led to the proposal that PriSLX may possess dual and sequential RNA and DNA synthetic abilities, perhaps akin to the DNA polymerase alpha assembly in eukaryotes [12].

## How does primase initiate synthesis?

**Figure 1.**
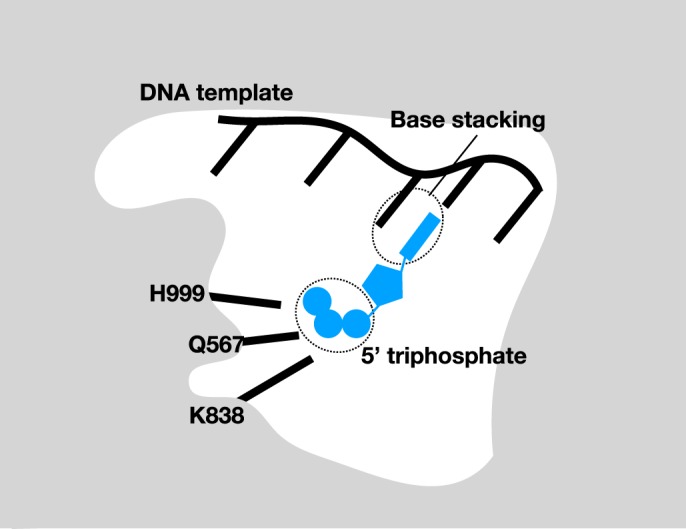
Model for how the bacterial RNA polymerase initiates RNA synthesis. The initiating nucleotide is stabilized by base pairing with the corresponding template base, partial base stacking with the preceding template base and coordination of the 5′-triphosphate with highly conserved residues in the polymerase active site. The consequence is that the nucleotide is positioned in a position analogous to the 3′-base of an elongating RNA chain. See reference [14] for further details.

**Figure 2.**
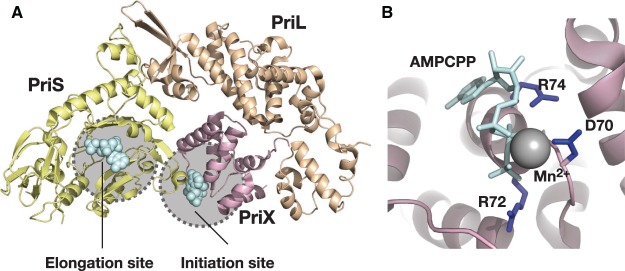
Two nucleotide-binding sites in the *Sulfolobus* primase. (**A**) Structure of *S. solfataricus* PriSLX assembly. PriS is in yellow and contains the polymerization elongation site, PriL is in wheat and PriX, which possesses the binding site for the initiating nucleotide triphosphate, is in pink. The ATP analog AMPCPP is shown in pale blue with atoms represented as spheres. (**B**) A close-up view of the initiation site in PriX — residues important for binding the triphosphate group of the pale blue AMPCPP directly or via a co-ordinated manganese ion (grey sphere) are shown in dark blue. See reference [13] for details. The figure was prepared using PDB File 5OF3.

The ability of primases to initiate nucleic acid synthesis *de novo* distinguishes them from the DNA polymerases. However, the cellular RNA polymerases are capable of initiating RNA synthesis without a primer. The structure of an initiation complex of RNA polymerase from the bacterium *Thermus thermophilus* revealed that the initiating nucleotide bound the same site as that occupied by the growing 3′-end of an elongating RNA chain [14]. However, the initiating nucleotide was stabilized by many unique interactions — including partial stacking with the preceding base on the single-stranded DNA template and interactions between the 5′-end signature triphosphate and highly conserved residues in the enzyme active site (Figure 1). Thus, these additional interactions enable the initiating nucleotide to mimic the 3′-end of a growing RNA chain. Intriguingly, biochemical studies of the eukaryotic DNA primase provided evidence for a highly distinct mode of initiation by primase [15,16]. More specifically, mutation of a highly conserved arginine residue in yeast or human primase led to a primase that could no longer initiate synthesis *de novo* but which was still capable of elongating a preformed oligoribonucleotide. This residue resided in a region of the primase highly distinct from the catalytic site in priS. Furthermore, this conserved residue was harbored in the C-terminal sub-domain of eukaryotic PriL that was related to the *Sulfolobus* PriX subunit. The crystal structure of the *Sulfolobus* PriSLX assembly bound to the ATP analog, AMPCPP, yielded novel insight into the role of this conserved region of primase (Figure 2). The structure revealed an extensive interface between PriS and PriL, as seen previously, and PriX occupying a space between the two larger subunits [13]. The N-terminal 47 amino acids of PriX were not resolved in the structure, nor was the C-terminal FeS-coordinating domain of PriL. Notably, biochemical assays revealed that these two regions of the PriL and PriX formed the primary interaction interface for PriX — suggesting a flexible mode of tethering PriX to the primase assembly. Importantly, AMPCPP was observed binding to two sites in PriSLX. One molecule was found in the polymerization active site of PriS and the second was bound to PriX. Furthermore, the PriX-bound AMPCPP was co-ordinated by the residue in PriX, Arginine 72 (R72), that was orthologous to the residue in eukaryotic PriL that was essential for initiation but not elongation. Subsequent biochemical experiments revealed that mutation of R72 to alanine abolished nucleotide interaction and that reconstituted PriSLX containing PriX R72A caused the primase to be initiation defective but elongation competent. Thus, this region of PriX in *Sulfolobus* and by inference, the conserved domain of PriL in eukaryotes, serves as the binding site for the initiating nucleotide. Interestingly, binding of nucleotide to PriX was exclusively mediated by the 5′-triphosphate moiety with R72 and its neighboring residues D70 and R74. Indeed, subsequent biochemical experiments revealed that PriX in isolation could not discriminate between nucleotides based on the identity of the base or even that of the sugar [17]. More specifically, PriX bound to either dATP or ATP with essentially identical affinities. However, it should be emphasized that in *Sulfolobus*, as in most cells, ribonucleotide triphosphate concentrations in the cell are ∼100-fold higher than the corresponding deoxyribonucleotide triphosphate [18]. Therefore, NTPs will be more likely to occupy the initiation site. Furthermore, the structure revealed that nucleotide binding by the PriS active site involved hydrogen bonding between the 2′-OH of the ribose and the peptide main chain — suggesting a basis for discrimination between NTPs and dNTPs in the elongation site [13]. Intriguingly, however, this discrimination seems to be on the basis of constraining nucleotide in an appropriate geometry for phosphodiester bond formation, rather than simply an affinity for substrate, as PriS actually has a higher affinity for dNTPs over NTPs but is less efficient and less processive at DNA synthesis [12,17]. The functional consequence is that when primase is presented with physiologically relevant concentrations of NTPs and dNTP, it incorporates the former to generate RNA primers [17].

Taken together, the structural and biochemical studies of PriSLX indicated that primase possesses two nucleotide binding sites — one essential for initiation, located within PriX, and the second site, in PriS, required for elongation. One, apparently paradoxical, feature of these findings is that the initiation and elongation sites in PriX and PriS, respectively, are over 30 Å apart. Clearly, a conformational alteration must be invoked to allow for juxtaposition of the initiation and catalytic sites. In support of this proposal, cross-linking studies that constrained the primase assembly in the ‘open’ conformation (as seen in the crystal structure) abrogated the enzyme's ability to initiate primer synthesis [13]. However, this conformationally constrained primase retained the ability to elongate preformed primers. Thus, the enzyme presumably ‘closes’ allowing initial dinucleotide condensation before re-opening as it proceeds into elongation mode. It may be significant in this regard that initial dinucleotide formation is the rate-limiting step in primer synthesis.

## Defining primer length

A unique feature of the initiating nucleotide during nucleic acid polymerization is that it retains its 5′-triphosphate moiety during chain elongation. This, therefore, serves as a molecular signature for the 5′-end. It is notable that this feature of the nucleotide is the principal determinant for its recognition by PriX. Indeed, this observation suggests a mechanism by which primase is able to define primer length. More specifically, if PriX retains its grip on the 5′-end of the growing chain, then the 5′-end defines the position of PriX while the 3′-end is located within the PriS active site. The primase could thus act as a caliper to define a maximal length of primer that could be accommodated between these two binding sites. In support of this, a crystal structure of C-terminal domain of human PriL (homologous to PriX) with a short RNA/DNA heteroduplex, reveals the interaction between the conserved arginine residue and the triphosphate of the 5′-end of the RNA chain [15]. A series of primer extension assays employing synthetic oligoribonucleotides with defined 5′-ends (5′-OH, monophosphate or triphosphate) with wild-type PriSLX and assembled heterotrimers containing mutated PriX, provided experimental evidence for this caliper model [17]. In addition to helping define primer length, the PriX — 5′-triphosphate interaction was also shown to be important for stabilizing the template-primer hybrid. This latter issue will be of particular importance in hyperthermophiles like *Sulfolobus* that grow at temperatures in excess of the melting point of the short RNA–DNA duplex. Clearly, a mechanism must exist whereby this duplex is stabilized prior to its transfer to the replicative DNA polymerase.

## A conserved mechanism for initiation in the archaeal/eukaryotic primase superfamily?

**Figure 3.**
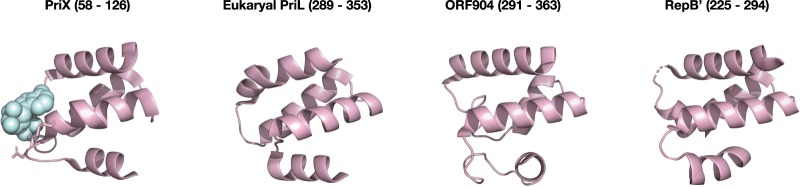
A comparison of the helical bundle regions of PriX, human PriL, ORF904 and RepB’. The initiating nucleotide bound to PriX is shown in pale blue. The figure was prepared from the co-ordinates in PDB files 5OF3, 5FOQ, 3MIM and 3H20.

The structural and functional relationship between eukaryotic PriL's CTD and PriX indicates that the initiation mechanism proposed above likely applies to primases in both domains of life. Interestingly, the Archaeal/Eukaryotic Primase (AEP) family extends to a broad range of extrachromosomal elements found in archaeal and bacterial organisms [10]. Furthermore, many eukaryotes encode PrimPol — a novel primase/polymerase involved in DNA damage tolerance — in which the catalytic center resembles the PriS catalytic site fold [19–21]. Many of these proteins contain a second domain in addition to the PriS-like catalytic domain (Figure 3). The structures of two such proteins have been determined, ORF904 from an archaeal plasmid, pRN1, and RepB' from the bacterial broad host range plasmid RSF1010 [22–24]. Strikingly, despite having essentially no sequence conservation, the accessory domains of both these proteins form helical bundles that bear considerable structural homology to PriX. In addition, biochemical studies have revealed that the helical bundle domain of both proteins is required for primer synthesis. In the case of ORF904, deletion of the helical bundle, while abrogating primase activity, did not prevent extension of a preformed primer by the isolated PriS-like catalytic domain [23]. A detailed study of the mode of action of ORF904 revealed that it primes with single riboucleotide that is then extended by DNA in the PriS-like elongation site. However, dNTPs could not be exploited to initiate synthesis by ORF904, suggesting that, unlike with PriX, the initiation site in ORF904 discriminates between deoxyribonucleotide and ribonucleotide triphosphates. Competition experiments revealed that binding of the initiating nucleotide was affected by modification of the 5′-triphosphate of the initiating nucleotide, indicating that, as in PriX, important contacts are made between the initiation site and this feature of the initiating nucleotide [25]. Taken together, the data suggest that the helical bundles of both ORF904 and RepB' are acting as the initiation sites for these extrachromosomal element primase/polymerases. However, it seems likely that fine details of the interaction between ORF904's helical bundle and initiating ribonucleotide will be distinct from analogous interface in PriX. Nevertheless, it seems probable that the existence of distinct polymerization and initiation sites extends across the AEP superfamily. Furthermore, if the helical bundles of these members of the extended AEP superfamily do indeed mediate initiation via interaction with the initiating nucleotide triphosphate, they may also play a role in primer length determination as proposed in the caliper model [17]. What then of the PrimPol enzyme? PrimPol has an N-terminal domain, related to the PriS fold [21], that is followed by a C-terminal domain. The C-terminal domain is predicted to bind zinc. Biochemical studies have revealed that, as with the helical bundles discussed above, the Zn-binding domain of PrimPol is required for initiation but not elongation [26]. Furthermore, the initiation activity of PrimPol is dependent on the 5′-triphosphate of the initiating nucleotide. Thus, it seems highly likely that this domain will mediate binding of the initiating nucleotide and its delivery to the catalytic site in the N-terminal domain. Currently, there is no structural information available for this domain of PrimPol, nor do structure prediction servers yield meaningful models. Nevertheless, it is tempting to speculate that the domain will adopt a helical bundle fold related to that seen in PriX, eukaryotic PriL and the extrachromosomal element primases described above.

## Abbreviations

AEP: Archaeal/Eukaryotic Primase
